# Evaluation of the Impact of Different Skeletal Orthodontic Anomalies on Condylar Asymmetry Using Cone-Beam Computed Tomography

**DOI:** 10.3390/diagnostics16050812

**Published:** 2026-03-09

**Authors:** Muhammet Bahattin Bingul, Seda Kotan, Saadet Cinarsoy Cigerim, Mevlude Yuce Polat

**Affiliations:** 1 Department of Oral and Maxillofacial Surgery, Faculty of Dentistry, Harran University, Sanliurfa 63300, Türkiye; 2 Department of Orthodontics, Faculty of Dentistry, Igdır University, Igdir 76000, Türkiye; dtsedakotan@gmail.com; 3 Department of Orthodontics, Faculty of Dentistry, Van Yuzuncu Yil University, Van 65080, Türkiye; saadetcinarsoy@live.com; 4 Department of Orthodontics, Faculty of Dentistry, Harran University, Sanliurfa 63300, Türkiye; drmevludepolat@gmail.com

**Keywords:** asymmetry, mandibular condyle, cone-beam computed tomography

## Abstract

**Background/Objectives**: This study aims to evaluate mandibular condylar asymmetry in individuals with different types of skeletal malocclusions using a three-dimensional imaging technique, and to determine the relationship between these anomalies and condylar asymmetry. **Methods**: The study included 100 individuals who visited the Department of Orthodontics Faculty of Dentistry between 2015 and 2020 and underwent Cone-Beam Computed Tomography (CBCT) imaging for various reasons. The evaluation of condylar asymmetry was performed using the Habets method, and measurements were carried out with the NemoCeph V.2017 software. Participants were categorized into skeletal Class I (2–4°), Class II (>4°), and Class III based on their ANB angles. For statistical analysis, frequency distribution, the Kruskal–Wallis H test, and Spearman’s correlation coefficient were used. **Results**: No statistically significant relationship was found between gender and skeletal classifications (*p* > 0.05). In terms of age, the mean age of individuals in the Class III group was significantly lower than that of those in the Class II group (*p* < 0.05). A weak positive correlation was observed between condylar and ramal indices in the overall sample (*p* = 0.029); however, this correlation was found to be moderate and statistically significant only within the Class III group (*p* = 0.002). **Conclusions**: The presence of a significant relationship between condylar and ramal asymmetries in individuals with Class III malocclusion indicates an increased risk of developing facial asymmetry if left untreated. These findings underscore the importance of skeletal malocclusions in influencing condylar morphology.

## 1. Introduction

Symmetry is defined as the appearance of an object that is equidistant from a specific axis, whereas asymmetry refers to a disruption of this balance. Although the human face generally appears symmetrical, it often contains certain degrees of asymmetry due to various anatomical and functional factors. Asymmetries originating from the mandibular condyle are typically subtle and difficult to detect, yet they have been reported in approximately 45% of the population [1]. Given that structural changes may adversely affect the function of the temporomandibular joint (TMJ), mandibular condylar asymmetry has been widely discussed in the literature [2,3]. Temporomandibular disorders (TMDs) play a significant role in the development of facial asymmetry, and occlusal asymmetry has been associated with skeletal facial asymmetry [4,5].

The etiology of condylar asymmetry can be influenced by both genetic and environmental factors. While it may be seen in association with joint disorders, developmental anomalies, or certain syndromes, condylar asymmetry can also occur in individuals with no syndromic conditions, normal occlusion, and otherwise healthy status [6]. Environmental factors such as prolonged occlusal interferences or chronic unilateral chewing habits may also contribute to the development of mandibular asymmetry [7]. Several methods have been used to evaluate condylar asymmetry. Techniques developed by Habets and Kjellberg assess condylar height to determine asymmetry levels, and these measurements have become more reliable with the use of modern three-dimensional imaging techniques such as cone-beam computed tomography (CBCT) (8,9). A study comparing the methods of Habets [8] and Kjellberg [9] reported similar consistency between the two approaches. In these methods, the condylar asymmetry index is assessed using panoramic radiographs and is calculated as the ratio of the difference in height between the right and left condyles to the sum of their heights [8].

Identifying the type of malocclusion in which condylar asymmetry is most commonly observed is essential for minimizing the risk of facial asymmetry and preventing treatment relapse in orthodontic patients. Understanding the prevalence of condylar asymmetry may also assist researchers in analyzing the etiology and morphological characteristics of each malocclusion. Although the impact of orthodontic malocclusions on mandibular morphology has been demonstrated in the literature, and some studies have reported statistically significant relationships between dental malocclusions and condylar asymmetry, studies specifically investigating the effects of skeletal malocclusions are limited [10]. Therefore, the present study aims to examine the influence of various skeletal orthodontic anomalies on condylar asymmetry.

## 2. Materials and Methods

This study included 100 patients who presented to the Department of Orthodontics between 2015 and 2020 and had undergone cone-beam computed tomography (CBCT) imaging for various reasons. Following the study design, ethical approval was obtained from the Non-Interventional Clinical Research Ethics Committee of Van Yüzüncü Yıl University (Decision No: 9 February 2021). The study was conducted in accordance with the principles of the Declaration of Helsinki. Individuals without severe facial deformities or occlusal asymmetry, with no neuromuscular disorders or systemic diseases, who had not previously received orthodontic treatment, and who showed no signs or symptoms of temporomandibular disorders were included. Additionally, subjects with no missing or extracted teeth (except third molars), no carious lesions or extensive restorations, no periodontal problems, no dental anomalies, and no cleft lip or palate were enrolled in the study. Skeletal malocclusions were determined using cephalometric radiographs, while condylar height and related measurements were obtained from CBCT images. For each selected patient, skeletal malocclusions in sagittal, vertical, and transverse dimensions were diagnosed along with Angle classification.

A Class I skeletal relationship was defined by an ANB angle between 2° and 4°, Class II by an ANB angle greater than 4°, and Class III by an ANB angle less than 2° [11,12,13].

### 2.1. Cephalometric Measurements

All lateral cephalometric radiographs were obtained using the same device and by the same operator, with patients in natural head position and lips at rest. The images were taken under standardized conditions using the Sirona Orthophos XG imaging system, Bensheim, Germany. Hard and soft tissue landmarks were identified, and measurements were made using the NemoCeph V.2017 software package. All analyses were performed on pretreatment lateral cephalometric radiographs.

### 2.2. CBCT Measurements

Patients who had CBCT scans taken for various orthodontic reasons (120 kV, 130 FOV, 0.300 voxel, 5 mA; KaVo 3D eXam, Biberach, Germany) were included in the study. CBCT images taken at the beginning of treatment were analyzed. All evaluations were performed by the same examiner (SK); each measurement was repeated three times, and the average was recorded. The Exam Vision software (version 2.0., ExamVision ApS, Copenhagen, Denmark) was used for measurements. To evaluate measurement reproducibility, intra-observer reliability was assessed. Twenty CBCT scans were randomly selected and re-evaluated by the same examiner two weeks after the initial measurements under identical conditions. Intraclass correlation coefficients (ICC) were calculated using a two-way mixed-effects model with absolute agreement. An ICC value above 0.75 was considered indicative of good reliability, and values above 0.90 were interpreted as excellent reliability.

Prior to measurement, all CBCT datasets were standardized to ensure reproducibility. Head orientation was determined using the Frankfort Horizontal Plane, aligned parallel to the horizontal axis in the sagittal view, as the primary reference. Then, a mid-sagittal plane was created and used for positional normalization to eliminate head rotation.

Multiplane reconstruction was performed to obtain consistent sagittal, coronal, and axial sections. Sagittal sections passing through the center of each condyle were selected based on maximum mediolateral width and clear visualization of cortical boundaries. Coronal and sagittal views were evaluated simultaneously to verify the identification of accurate reference points.

All anatomical reference points were defined according to predefined criteria, and measurements were performed under standard imaging conditions to minimize variability related to orientation.

**Figure 1:**
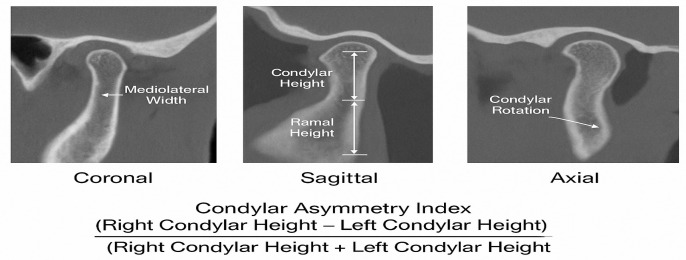
The schematic illustration (OpenAI, GPT-5.2). Morphological assessment of the condyle and calculation of the condylar asymmetry index in coronal, sagittal and axial CBCT sections.

In the evaluation process, anatomical landmarks were identified and lines were drawn on 3D images according to the method developed by Habets and colleagues. Although the Habets index was originally developed for panoramic radiographs, it has been widely applied in three-dimensional CBCT analyses due to its proportional linear approach, which is unaffected by magnification or projection distortion. In this study, standardized sagittal reconstructions from CBCT were used to minimize projection artifacts and improve measurement accuracy. The highest points of the condyles and the most posterior points of the rami on both sides of the mandible were marked. A horizontal reference line (X line) was drawn through the posterior points, and a vertical line (Y line) was drawn from the most superior point of each condyle perpendicular to the X line. The distance between the intersection point of these lines and the posterior point was measured as the condylar height. The condylar asymmetry index was then calculated using the formula proposed by Habets et al.: Condylar Asymmetry Index = [(Right − Left)/(Right + Left)] × 100 [8]. A schematic illustration used in this study was generated with the assistance of ChatGPT (OpenAI, GPT-5.2) (Figure 1).

### 2.3. Statistical Analysis

To determine the descriptive characteristics of the participants, frequency analysis was used. The normality of the distribution of numerical variables was assessed using the Shapiro–Wilk test for groups smaller than 50 and the Kolmogorov–Smirnov test for groups larger than 50. Relationships between non-normally distributed variables were analyzed using Spearman’s rho correlation coefficient. For comparisons of non-normally distributed values according to categorical demographic variables, the Kruskal–Wallis H test was applied. Given the non-normal distribution of condylar and ramal measurements, median (Min–Max) values were prioritized for descriptive statistics. Mean ± SD values are presented for general reference only, ensuring alignment with nonparametric inferential tests (Kruskal–Wallis H test). In cases of multiple comparisons, Bonferroni-corrected Dunn’s test was used. Quantitative data were reported as mean ± standard deviation (Mean ± SD), median, minimum, and maximum (Med (Min–Max)), while categorical data were expressed as frequency (*n*) and percentage. A *p*-value of <0.05 was considered statistically significant in all analyses and interpretations. Normality was assessed using the Shapiro–Wilk test for groups with *n* < 50 and the Kolmogorov–Smirnov test for groups with *n* ≥ 50. In addition, skewness and kurtosis values were examined, and distributions with values between −1.5 and +1.5 were considered approximately normal. Data were analyzed using IBM SPSS version 27 software (IBM Corp Armonk NY, USA, Released 2020).

## 3. Results

This study included 100 patients who presented to the Department of Orthodontics at Van Yüzüncü Yıl University Faculty of Dentistry between 2015 and 2020 and underwent CBCT imaging for various reasons. Table 1 presents the demographic and clinical characteristics of the participants. Regarding gender distribution, 41% of the participants were male, and 59% were female. The distribution of skeletal malocclusion classes was 34% for Class I, 46% for Class II, and 20% for Class III. The mean age of the participants was 22.16 years, with a standard deviation of 4.822.

When physiological measurements were examined, the mean right and left condylar heights were 8.005 mm and 7.968 mm, respectively. The mean right and left ramus heights were 40.98 mm and 40.666 mm, respectively. The average values for the condylar index and ramal index were 5.6 and 2.08, respectively.

The intra-observer reliability analysis demonstrated excellent agreement for all linear measurements. The ICC values were 0.93 for condylar height and 0.91 for ramus height measurements, indicating high reproducibility and minimal measurement error.

Table 2 presents the results regarding the relationship between gender and skeletal class groups among the participants. According to these results, no statistically significant association was found between gender and skeletal classification (*p* > 0.05).

Table 3 presents the comparative results of certain characteristics of the participants based on their skeletal classification groups. According to the findings, there was no statistically significant difference in condylar index and ramal index values among the skeletal classes (*p* > 0.05). However, a statistically significant difference was observed in the age values across the groups (*p* < 0.05). Upon further examination, it was found that participants in the Class III group were significantly younger than those in the Class II group.

Table 4 presents the results of the relationship between condylar index and ramal index values. A weak but statistically significant positive correlation was observed between the condylar and ramal index values among all participants (*p* < 0.05). When analyzed by group, no statistically significant correlation was found between these indices in Class I and Class II participants (*p* > 0.05). However, a moderate and statistically significant positive correlation was identified in the Class III group (*p* < 0.05).

All continuous variables are presented with median (Min–Max) values as primary descriptive statistics due to non-normal distributions. Mean ± SD values are reported for general reference. Kruskal–Wallis H tests revealed no statistically significant differences in condylar or ramal asymmetry indices among skeletal classes (*p* > 0.05). A moderate positive correlation between condylar and ramal indices was observed only within the Class III subgroup (r = 0.656, *p* = 0.002).

## 4. Discussion

In daily life, individuals often fail to notice facial asymmetries, and such conditions are generally considered normal. However, these asymmetries can be detected through radiographic evaluations. While moderate facial asymmetries can be managed with various orthodontic and orthopedic approaches, severe asymmetries may require a combination of orthodontic treatment and surgical intervention [14].

Recent studies evaluating facial asymmetry have largely focused on the mandible [15]. In a study by Sato et al., it was reported that 34% of patients requiring orthodontic treatment exhibited facial asymmetry, and 74% of these cases showed mandibular asymmetry [16]. Mandibular asymmetry has been attributed to abnormal unilateral active growth of the condyle, resulting in excessive length and volume on the affected side [5,17].

It has been demonstrated that orthodontic malocclusions have a significant impact on mandibular condylar morphology. Asymmetric function and activity of the jaws can lead to differential development between the right and left sides of the mandible [10]. In cases of unilateral posterior crossbite, asymmetric muscle function and condylar height have been reported [10,18]. While some studies have found no clear association between asymmetry and dental malocclusion, others have reported varying results regarding the relationship between condylar asymmetry and dental malocclusion [2,10,18,19].

Previous studies have shown that patients with different skeletal malocclusions exhibit varying incidences of mandibular asymmetry. Identifying the most common malocclusion type associated with condylar asymmetry is important to prevent relapse and minimize the risk of developing facial asymmetry in orthodontic patients. Therefore, the present study aimed to investigate this issue in greater detail.

Due to the limitations of two-dimensional analyses, three-dimensional cone-beam computed tomography (CBCT) imaging is recommended. There are concerns regarding the reliability of panoramic radiographs in predicting mandibular asymmetry, especially due to the influence of vertical mandibular growth [20]. Moshfeghi et al. reported that CBCT images in axial and coronal planes provide high accuracy and that the obtained linear measurements are reproducible in the maxillofacial region [21]. Our CBCT-based measurements provide more accurate and volumetrically reliable assessments of condylar and ramal asymmetry compared to studies relying on panoramic radiographs, which are subject to projection errors and underestimation of vertical discrepancies. This methodological difference may explain certain discrepancies between our findings and prior literature reporting higher asymmetry indices in similar skeletal classes. In our study, no statistically significant correlation was found between condylar and ramal indices among participants in Class I.

CBCT allows clinicians to directly observe the anatomical regions where asymmetries occur in the maxillofacial area, thereby enhancing diagnostic accuracy and reproducibility in clinical research [15]. Accordingly, CBCT was utilized in this study to obtain more accurate and reliable measurements.

The voxel size used in the present study was 0.300 mm, which directly influences spatial resolution and landmark localization accuracy in CBCT imaging. Smaller voxel sizes generally improve spatial resolution; however, they may increase radiation exposure and image noise. Previous studies have demonstrated that CBCT-derived linear measurements obtained with voxel sizes in the range of 0.2–0.3 mm show high accuracy, with measurement errors typically within ±0.2–0.3 mm in the maxillofacial region [21].

Nevertheless, partial volume effects may occur when anatomical structures approach the voxel dimension, potentially influencing the precision of small linear differences. Therefore, minor asymmetry index variations should be interpreted cautiously, particularly when differences fall within the expected measurement error range.

Various methods have been used to evaluate condylar asymmetry. A study assessing the methods of Habets and Kjellberg found both techniques to have similar reliability [8,9]. The Habets method can be used in patients with temporomandibular joint dysfunction and different skeletal and occlusal patterns [22]. In our study, the Habets technique was employed to evaluate the measurements in Angle Class I, Class II, and Class III occlusal groups. Although the Habets index was originally designed for two-dimensional radiographs, the use of standardised sagittal reconstructions derived from CBCT reduces projection-related artefacts. However, future studies using fully three-dimensional volumetric analyses may provide a more comprehensive assessment of condylar asymmetry.

The results of the study by Alkış et al. were consistent with those of Kasimoğlu et al., who reported no significant relationship between occlusion type and condylar asymmetry in Class I, II, and III patients [20,23]. However, another study by Al Taki et al. reported significantly greater condylar asymmetry in Class II patients compared to those in Class I [24].

In our study, no statistically significant correlation was found between condylar and ramal indices among participants in Class I, Class II, or Class III groups (*p* > 0.05), which is consistent with the literature. These non-significant intergroup differences may be attributable to methodological factors, including sample size limitations, retrospective design, and the inherent variability in individual condylar morphology. Furthermore, the use of standardized CBCT multiplanar reconstructions, while reducing measurement error, may capture subtle asymmetries that differ from those reported in two-dimensional analyses, highlighting potential sources of discrepan. However, a moderate and statistically significant positive correlation was observed between these indices in the Class III group (*p* < 0.05). There is currently no specific literature available to directly compare this result. Age-related variation in skeletal development may influence condylar asymmetry indices, particularly in younger individuals whose mandibular growth has not yet ceased. In our sample, a statistically significant difference in age was observed among the skeletal classes (*p* < 0.05), with Class III participants being younger than those in Class II. Given that mandibular growth continues into late adolescence, this age discrepancy may partially account for the moderate correlation observed between condylar and ramal indices in the Class III group. Therefore, some of the observed asymmetry may reflect ongoing growth dynamics rather than skeletal classification alone. The significantly lower mean age observed in Class III group may affect asymmetry findings because mandibular growth continues into late adolescence. Therefore, part of the observed correlation may reflect ongoing growth dynamics rather than skeletal classification alone. This growth-related variability suggests that part of the observed asymmetry may reflect ongoing mandibular development rather than skeletal classification alone. Accounting for age and growth stage is therefore essential in interpreting condylar and ramal asymmetry indices. Future studies using multivariate regression models controlling for age and gender are recommended.

A review of the literature indicates that the mean condylar asymmetry index values reported in different studies are higher than 3% in both Class I and Class III occlusion groups, which is consistent with the findings of our study [2,23]. Although most studies in the literature have not specifically examined the relationship between asymmetry and gender, those that have investigated the association between gender and condylar or ramal asymmetries reported no statistically significant relationship. Similarly, our study found no association between asymmetry and age, consistent with the existing literature.

In a study evaluating the relationship between vertical mandibular condylar asymmetry and Angle Class I, II, and III malocclusions, as well as unilateral posterior crossbite, it was reported that patients with posterior crossbite presented with asymmetric condylar heights and may be at risk for developing skeletal mandibular asymmetry [20]. However, Uysal et al. found no significant differences between individuals with normal occlusion, unilateral posterior crossbite, and bilateral posterior crossbite [22]. Lee et al. reported that individuals with asymmetric skeletal Class III malocclusion showed significant differences in condylar height, ramus height, and the posterior portion of the mandibular body compared to individuals with normal occlusion [25].

Studies using panoramic radiographs to investigate condylar asymmetry have stated that asymmetry index values greater than 3% should be considered indicative of vertical asymmetry. In the study by Kasimoğlu et al., the condylar asymmetry indices were reported as 6.51 ± 5.39 in Class I, 6.23 ± 7.19 in Class II, and 7.77 ± 6.51 in Class III, suggesting the presence of asymmetry “[20]”. Similarly, our study found the condylar asymmetry indices to be 5.911 ± 7.989 in Class I, 6.214 ± 6.261 in Class II, and 3.663 ± 3.798 in Class III. The lower value in the Class III group may be explained by the unbalanced growth of the mandible caused by excessive growth on one side, and this finding may also be attributed to the use of CBCT in our study. CBCT enhances the accuracy of diagnosis and clinical research by providing detailed visualization of the anatomical areas where maxillofacial asymmetries occur. In this study, using NemoCeph software, landmarks were identified on CBCT images, and measurements were performed, yielding more reliable results compared to traditional two-dimensional radiographs. Mendoza et al. also reported that the prevalence of asymmetry indices based on tomographic images was at least 2.5 times higher for all malocclusion types, supporting the reliability of CBCT-derived asymmetry measurements [26].

Identifying the malocclusion type most commonly associated with condylar asymmetry is critical for minimizing the risk of developing facial asymmetries and for effective orthodontic follow-up. Knowing the prevalence of condylar asymmetry can also guide researchers in analyzing the etiology and morphological characteristics of each malocclusion type.

The findings of this study indicate a more pronounced relationship between condylar and ramal asymmetry in individuals with skeletal Class III malocclusion. This may be attributed to imbalanced mandibular growth. Previous studies have reported that mandibular prognathism is associated with increased asymmetry [25].

While our findings suggest that individuals with Class III malocclusion may exhibit more pronounced condylar and ramal asymmetry, these results should be interpreted as exploratory. The data support enhanced diagnostic awareness rather than providing definitive guidance for treatment planning, and further longitudinal studies are required to establish predictive clinical implication. Functional imbalances, such as habitual unilateral chewing, are known to have long-term effects on condylar morphology [22]. Therefore, morphological analysis using CBCT during the growth and development period can enhance both diagnostic and therapeutic outcomes.

Furthermore, clinical experience suggests that treatment success in Class III individuals depends on early interventions aimed at redirecting mandibular growth. The use of functional appliances can help modify the direction of mandibular development and reduce the asymmetric effects on condylar growth. Early intervention can support symmetrical development of the facial skeleton and potentially reduce the need for surgical treatment [24].

CBCT offers three-dimensional detail in the assessment of condylar asymmetry, revealing not only morphological structures but also joint positioning and its relationship with surrounding tissues. This makes it possible to differentiate between growth-related and functional asymmetries, allowing for the development of individualized treatment plans.

However, the frequent use of panoramic radiographs in clinical practice presents limitations due to their lower accuracy compared to CBCT. In cases with suspected facial asymmetry, CBCT is strongly recommended for orthodontic treatment planning due to its significant advantages [26].

Despite providing valuable insights, the present study has certain limitations. First, it employed a retrospective design, which inherently limits the strength of causal inferences due to its observational nature. Second, the sample size was limited to 100 patients, which restricts the generalizability of the findings compared to larger, more diverse study populations. Although the Habets asymmetry index was originally developed for panoramic radiographs, it is mathematically based on proportional linear differences between the right and left sides rather than absolute magnified distances. While panoramic imaging is affected by projection distortion, CBCT provides distortion-free volumetric data. In this study, standardized multiplanar reconstructions were aligned using reference planes to ensure consistent measurement orientation across subjects. Thus, the asymmetry index was calculated under geometrically controlled conditions within the CBCT environment.

However, linear asymmetry indices represent a simplified two-dimensional assessment of a three-dimensional structure. Fully volumetric morphometric analyses may offer a more comprehensive evaluation of condylar asymmetry and should be considered in future studies.

No multivariable regression was performed; thus, potential confounders such as age, sex, and vertical skeletal pattern were not adjusted. Notably, the Class III subgroup was younger than Class II, which may have influenced the observed condylar and ramal asymmetry. Therefore, associations between skeletal classification and condylar or ramal asymmetry indices should be interpreted as correlations rather than causal relationships. Mechanistic inferences cannot be drawn from these data. Future studies should use multivariate models to better isolate the effects of skeletal malocclusion.

Moreover, the analyses based on skeletal classes did not account for potential interactions between demographic variables such as age and gender. Future studies should consider these factors in greater depth.

## 5. Conclusions

Within the study cohort, no statistically significant differences in condylar or ramal asymmetry indices were observed across skeletal classes. Within individuals presenting with skeletal Class III malocclusion, a synergistic relationship was observed between condylar and mandibular ramus asymmetry. In particular, individuals with Class III malocclusion exhibited more pronounced asymmetric condylar development. These findings highlight the importance of considering skeletal classification during the orthodontic assessment process. The observed association between condylar and ramal asymmetries in Class III individuals highlights the importance of diagnostic evaluation of skeletal asymmetry. However, given the exploratory nature of this study and the moderate effect sizes, these findings should not be interpreted as definitive predictors of clinical outcomes. Overall, the results emphasize the critical influence of skeletal malocclusions on condylar morphology.

## Figures and Tables

**Table 1:** Demographic characteristics and condylar and ramal measurement parameters of the study sample.

Variable	n	Median (Min–Max)	Mean ± SD
Age	100	22 (13–55)	22.16 ± 4.82
Right condyle (mm)	100	7.92 (4.29–12.5)	8.005 ± 1.745
Left condyle (mm)	100	7.92 (4.28–12.76)	7.968 ± 1.753
Condylar index	100	3.34 (0.073–32.157)	5.60 ± 6.54
Right ramus (mm)	100	40.45 (33.27–49.51)	40.98 ± 3.59
Left ramus (mm)	100	40.38 (34.63–51.5)	40.66 ± 3.27
Ramal index	100	1.71 (0.024–7.414)	2.08 ± 1.57

**Table 2:** Comparison of Participants’ Gender Across Skeletal Classification Groups.

Gender/Class	Class			χ^2^	*p*
	1	2	3		
Male	13 (31.7%)	18 (43.9%)	10 (24.4%)	0.844	0.656
Female	21 (35.6%)	28 (47.5%)	10 (16.9%)		

χ^2^: Chi-square test for independent samples. Participant data are presented as n (%).

**Table 3:** Comparison of Selected Characteristics of Participants Across Skeletal Classification Groups.

Variable	Class I	Class II	Class III	Test	*p*
Age	22 (14–28)	23 (18–55)	21 (13–28)	KW H = 7.269	0.026
Condylar index	3.24 (0.073–32.158)	4.31 (0.084–28.543)	2.06 (0.274–15.959)	KW H = 0.839	0.226
Ramal index	1.82 (0.407–4.430)	1.80 (0.037–7.415)	1.44 (0.025–5.343)	KW H = 0.354	0.443

KW: Kruskal–Wallis H test, *p*: Significance value.

**Table 4:** Relationship Between Condylar Index and Ramal Index.

Group	Relationship	Correlation/Significance	Value
Overall	Condylar Index–Ramal Index	*s*	0.218
		*p*	0.029
Class 1	Condylar Index–Ramal Index	*s*	0.007
		*p*	0.968
Class 2	Condylar Index–Ramal Index	*s*	0.282
		*p*	0.057
Class 3	Condylar Index–Ramal Index	*s*	0.656
		*p*	0.002

*s*: Scheme Spearman correlation coefficient, *p*: Significance value.

## Data Availability

The datasets generated and/or analyzed during the current study are available from the corresponding author on reasonable request.

## Abbreviations

The following abbreviations are used in this manuscript:

CBCT	Cone-beam computed tomography
TMJ	Temporomandibular joint
